# Annotations

**Published:** 1907-02-09

**Authors:** 


### ANNOTATIONS.

### The Late Sir Michael Foster.

The death of Sir Michael Foster will be generally recognised as a severe loss to the position and influence of science considered as a factor in the development of national life and welfare. If the lapse of time and other influences had removed him, at least in his special sphere, from the ranks of those engaged in original research, he still remained, even though no longer a teacher in the technical sense of that term, one of the most energetic and consistent preachers of the claims of science, and of the need for the recognition of these in the efficient organisation of national life. Only a few days prior to his death, when speaking at the Mansion House in support of the British Science Guild, he was enforcing this essential doctrine, and was saying "science is not for men of science alone." In this direction in particular, the loss of his interest and influence will be severely felt. For, however it is to be explained, a large section of the British public—and the governing classes contribute largely in this direction—cultivates a distrust or even an active dislike of science and its defenders. An incident towards the close of Sir Michael Foster's own career illustrates this statement. As most people know, Foster taught physiology at Cambridge for some thirty years, and his reputation and influence were among the proudest boasts of the medical school of the University. Yet when, on his retiral in 1903, an attempt was made to recognise his great services by the grant of a pension and by a proposal to elect him to an extraordinary professorship, both suggestions excited a most active and energetic opposition. His long record as a scientific worker and the charm of his literary style have made him familiarly known to many who never saw his face, whilst his grateful pupils are to be found in all lands. To the last he was keenly interested in the work of the Royal Commission on Tuberculosis, of which he was appointed Chairman in 1901.

### Angioneurosis.

Vasomotor symptoms arising from a morbid condition of the sympathetic system are of a somewhat indefinite and very varying nature; they have, consequently, often suffered neglect at the hands of clinical teachers. Many of these manifestations are rather indications of an instability of the sympathetic system than of any actual morbid condition. Nevertheless it is well that we should have clear ideas on these neuroses, and Dr. Thomas D. Savill has filled a gap in our system by defining these conditions and their special features. He points out that these vasomotor symptoms of angioneurosis present four special clinical characteristics. They are paroxysmal; they are more common in women than in men, and especially in emotional women; they tend to recur in some form or another throughout life, and when localised they tend to be bilateral and symmetrical. Among these vasomotor disturbances Dr. Savill would place nervous faintings, flush-storms and ischæmic storms, often followed by the passage of large quantities of pale, watery urine, which he ascribes to a sudden dilation of the splanchnic vessels. Circulatory disturbances, such as palpitation and rapid pulse, and pulmonary disturbances, including asthma, also belong to the same category, and he suggests that migraine and even epilepsy may have a vasomotor origin. Many skin eruptions are of a vasomotor nature, and it is especially necessary that these should be carefully distinguished and diagnosed for purposes of treatment. The sovereign remedy for all these conditions indicating an irritability of the reflex centres is bromine. Other remedies are also indicated, according as the symptoms point to a condition of angiospasm or vascular dilatation.

### Effect of a Meat Diet on Animals.

Some very interesting investigations are being conducted in the physiological laboratory of the University of Edinburgh with a view to ascertain the effects of a meat diet on animals and their progeny, and Dr. D. Chalmers Watson recently read a paper before the Pathological Society on the results so far obtained. The objects of these researches were to ascertain what organs, if any, are specially stimulated by a meat diet; to throw light upon doubtful questions of dietetic treatment, and to determine whether an increase in the consumption of meat food is in any way connected with the increasing incidence of certain diseases. Rats were used, and sets of controls were fed on bread and milk. Rats fed exclusively on meat showed marked retardation of growth, and their reproductive powers were diminished. The second generation of meat-fed rats gave a high mortality. The meat diet, when commenced at the age of two to four months, was prejudicial to the occurrence of pregnancy, and also to lactation, owing to the smaller development of mammary tissue. The thyroid glands showed in the majority of cases marked changes, suggesting that the activity of the gland was at first stimulated by the diet and later exhausted. The kidneys also showed changes due to excessive strain on their power of excretion. As pointed out by Dr. E. I. Spriggs and Dr. Hale White, some of the phenomena observed might be due rather to the absence of certain necessary food constituents, such as calcium salts, from the meat diet, than to any direct pernicious effect of the meat itself, and it is to be hoped that by making certain variations in the diet in the further investigations which we understand are still in progress, Dr. Chalmers Watson may be able to draw more definite conclusions as to the actual influence of the meat *per se* on the animals. The physician is still very much in the dark as to the effects of the different foodstuffs on the metabolic processes of the body, especially in the many morbid conditions of a chronic nature which he is called upon to treat. Any investigations, therefore, which are likely to throw light on these questions are of great interest and importance; but it must always be borne in mind that it is not justifiable to draw inferences in regard to man directly from such experiments upon animals.

